# Rapid modification of the bone microenvironment following short-term treatment with Cabozantinib *in vivo*

**DOI:** 10.1016/j.bone.2015.08.003

**Published:** 2015-12

**Authors:** Marie-Therese Haider, Keith D. Hunter, Simon P. Robinson, Timothy J. Graham, Eva Corey, T. Neil Dear, Russell Hughes, Nicola J. Brown, Ingunn Holen

**Affiliations:** a Department of Oncology, University of Sheffield, Sheffield, UK; b School of Clinical Dentistry, University of Sheffield, Sheffield, UK; c CR-UK Imaging Centre, Division of Radiotherapy and Imaging, The Institute of Cancer Research, Sutton, Surrey, UK; d Department of Urology, University of Washington Medical Center, Seattle, WA, USA; e South Australian Health and Medical Research Institute, Adelaide, SA, Australia

**Keywords:** CBZ, Cabozantinib, GFP Ob^+^, green fluorescent protein expressing cells of the osteoblastic lineage, CTRL, control, PBS, phosphate buffered saline, TRAP, tartrate resistant alkaline phosphatase, PINP, procollagen type 1 N-terminal propeptide, MET, hepatocyte growth factor receptor, VEGFR-2, vascular endothelial growth factor receptor 2, RTK, receptor tyrosine kinase, HGF, hepatocyte growth factor, VEGF, vascular endothelial growth factor, Bone microenvironment, Osteoblast, Osteoclast, Bone marrow, Vasculature

## Abstract

**Introduction:**

Bone metastasis remains incurable with treatment restricted to palliative care. Cabozantinib (CBZ) is targeted against multiple receptor tyrosine kinases involved in tumour pathobiology, including hepatocyte growth factor receptor (MET) and vascular endothelial growth factor receptor 2 (VEGFR-2). CBZ has demonstrated clinical activity in advanced prostate cancer with resolution of lesions visible on bone scans, implicating a potential role of the bone microenvironment as a mediator of CBZ effects. We characterised the effects of short-term administration of CBZ on bone in a range of *in vivo* models to determine how CBZ affects bone in the absence of tumour.

**Methods:**

Studies were performed in a variety of *in vivo* models including male and female BALB/c nude mice (age 6–17-weeks). Animals received CBZ (30 mg/kg, 5 × weekly) or sterile H_2_O control for 5 or 10 days. Effects on bone integrity (μCT), bone cell activity (PINP, TRAP ELISA), osteoblast and osteoclast number/mm trabecular bone surface, area of epiphyseal growth plate cartilage, megakaryocyte numbers and bone marrow composition were assessed. Effects of longer-term treatment (15-day & 6-week administration) were assessed in male NOD/SCID and beige SCID mice.

**Results:**

CBZ treatment had significant effects on the bone microenvironment, including reduced osteoclast and increased osteoblast numbers compared to control. Trabecular bone structure was altered after 8 administrations. A significant elongation of the epiphyseal growth plate, in particular the hypertrophic chondrocyte zone, was observed in all CBZ treated animals irrespective of administration schedule. Both male and female BALB/c nude mice had increased megakaryocyte numbers/mm^2^ tissue after 10-day CBZ treatment, in addition to vascular ectasia, reduced bone marrow cellularity and extravasation of red blood cells into the extra-vascular bone marrow. All CBZ-induced effects were transient and rapidly lost following cessation of treatment.

**Conclusion:**

Short-term administration of CBZ induces rapid, reversible effects on the bone microenvironment *in vivo* highlighting a potential role in mediating treatment responses.

## Highlights

• Short-term administration of Cabozantinib (30 mg/kg) induces rapid alterations of the bone microenvironment *in vivo*.
• Cabozantinib reduced the number of osteoclasts, increased trabecular thickness as well as osteoblast numbers.
• Rapid alterations in the epiphyseal growth plate and bone marrow cellular composition were observed compared to control.
• The Cabozantinib-induced effects on the bone microenvironment were rapidly reversible on cessation of treatment.
• Therapeutic effects of Cabozantinib may be mediated in part through cells of the bone microenvironment.

## 1 Introduction

Effective suppression of bone metastasis requires therapeutic targeting of both the tumour and the bone microenvironment, hence use of agents with multiple targets may provide an opportunity to improve outcome for patients with skeletal metastases. Cabozantinib (CBZ) is a receptor tyrosine kinase (RTK) inhibitor with potent activity against multiple RTKs, including VEGFR-2 and MET, that mediate tumour survival, metastasis and angiogenesis and are also expressed by a number of cell types in bone [1], [2], [3], [4]. This profile suggests that CBZ has potential as an anti-tumour agent for use in patients with bone metastasis.

Pre-clinical studies in models of prostate cancer bone metastasis demonstrated a decrease in tumour volume, tumour necrosis, suppression of tumour growth in addition to altered bone remodelling following long-term CBZ treatment, suggesting tumour cells as well as cells of the bone microenvironment as potential cellular targets of CBZ [5], [6]. A phase II randomized discontinuation trial in patients with advanced prostate cancer demonstrated clinical activity, including increased resolution in bone scans in addition to pain relief in more than 60% of evaluable patients [7], [8], [9]. Both clinical and preclinical studies therefore suggest the bone microenvironment as a potential mediator of observed treatment responses. However, it is still unclear whether CBZ targets bone metastases directly, indirectly through modulating the bone, or both.

Bone metastasis involves complex crosstalk between the tumour cells and cells of the bone microenvironment including osteoclasts, osteoblasts, haematopoietic and vascular cells. Bone turnover is regulated through receptor activator of nuclear factor-kappa-B (RANK) and RANK ligand (RANKL) interactions. Osteoclast differentiation and maturation is mediated by binding of RANKL (expressed by osteoblasts) to RANK (expressed by osteoclasts) [10]. In addition to RANK/RANKL signalling, the MET and VEGF signalling pathways regulate the tightly balanced coupling between osteoblasts and osteoclasts, as both cell types express target receptors [2], [3], [4], [11] and are therefore potentially affected by CBZ. CBZ is a multiple tyrosine kinase inhibitor, also targeting Ret, Kit, Flt-1/3/4, Tie2, and AXL, all of which might be involved in bone remodelling as well as bone cell biology [12], [13]. In this study we have focussed on exploring the potential role of MET and VEGFR in the bone effects of CBZ.

The role of VEGF in promoting tumour angiogenesis and promoting tumour cell survival are well established, but VEGF/VEGFR signalling also plays a pivotal role in ossification (reviewed in [14]) and in maintaining the balance between bone formation and resorption by regulating osteoblast and osteoclast survival and activity [15]. Crosstalk between osteoblasts and haematopoietic stem cell niches are suggested (reviewed in [16]); however, how communication between vascular endothelial cells and bone cells is mediated remains to be established. CBZ could therefore inhibit the VEGF-driven processes of vascular remodelling and bone turnover, both associated with tumour growth and progression in bone.

Hepatocyte growth factor (HGF) is the only known ligand for the RTK MET. MET/HGF signalling is instrumental in embryogenesis, cell proliferation, motility and survival in addition to angiogenesis and wound healing [17], [18], [19], [20]. High levels of MET in cancer are associated with poor prognosis and are known to promote tumour invasion, metastasis, growth and survival [21]. In addition to expressing MET, osteoblasts and osteoclasts also secrete HGF indicating that the HGF/MET signalling axis regulates growth, activity and survival of these cells through both, autocrine and paracrine mechanisms [2], [22], [23]. In addition HGF is secreted by cells of mesenchymal origin [24] including bone marrow stromal cells [25]. A number of cell types that play a role in bone turnover, as well as in metastasis express target receptors of CBZ. This includes haematopoietic cells like megakaryocytes, which in turn are demonstrated to modify both osteoblasts and osteoclasts *in vitro* [26], [27]. When co-cultured with megakaryocytes, increased osteoblast proliferation and decreased osteoclast formation as well as activity has been observed [26], [28]. Cartilage-synthesizing chondrocytes express receptors for MET and VEGF and are therefore also potential targets of CBZ [4], [29], [30], [31]. In addition, HGF induces proliferation and migration of endothelial cells *in vitro* as well as angiogenesis *in vivo* [19]. Taken together, these published reports support that CBZ has the capacity to modify a number of cell types in the bone microenvironment that contribute to both normal bone homeostasis and cancer-induced bone disease *via* the targeting of MET.

The involvement of MET and VEGFR signalling in bone remodelling and metastasis offers the opportunity for therapeutic targeting of the bone microenvironment in addition to the cancer itself. To date, most studies of CBZ have focused on advanced cancer-induced bone disease [5], [32], [33], but the extensive loss of bone, combined with the profound effects of increasing tumour burden in this setting, masks the effects of therapies on the bone microenvironment. Indeed, initial observations on non-tumour bearing bone in mice [32], [33] indicate that CBZ may have direct effects on the bone microenvironment. It is therefore important to fully elucidate the effects of CBZ on bone in the absence of tumour.

We have determined the short-term treatment effects of CBZ on the tumour-free bone microenvironment *in vivo* using a range of models, including contralateral non-tumour bearing tibiae from mice with established prostate cancer-induced bone metastasis following long-term treatment with CBZ. To our knowledge this is the first comprehensive study to demonstrate that treatment effects observed in models of bone metastasis might be partially mediated by cells of the bone microenvironment.

## 2 Methods

### 2.1 *In vivo* experiments

All *in vivo* experiments were performed in compliance with the UK Animals (Scientific Procedures) Act 1986 and were reviewed and approved by the local Research Ethics Committee of the University of Sheffield (Sheffield, UK). Work was performed under UK Home Office regulations (project license 40/3531, personal license 40/10913).

### 2.2 Animal models

To allow comparison of the effects of CBZ in different bone microenvironments, studies were performed in animal models of different strain, sex and age as follows: 1) 6-week old male BALB/c nude mice, 2) 6-week old female BALB/c nude mice (both Charles River, UK), 3) 8–9-week old female genetically engineered mice expressing GFP-positive cells of the osteoblastic lineage on a BALB/c nude background ((BALB/cAnNCrl.Cg-Tg(Col1a1-GFP)Row Foxn1^nu/nu^, described in [34], heterozygous, referred to as GFP Ob^+^ mice) and 4) 17-week old female GFP Ob^+^ mice (both Leeds Institute for Molecular Medicine, UK).

In murine models, bone remodelling is reduced with increasing age. We therefore established effects of CBZ in young (8–9 week old) mice with high bone turnover in addition to older (17-week old) mice with lower bone turnover.

### 2.3 Drug treatment and sample collection

Cabozantinib (XL184), a kind gift from Exelixis Inc., South San Francisco, California, USA, was prepared in sterile filtered H_2_O. To aid dissolution of the drug 5 μL 1 N HCl per 3 mg/mL CBZ were added according to company recommendations and administered by oral gavage. The drug suspension was prepared fresh on the day of administration. To analyse effects of CBZ on the bone microenvironment, animals were randomized in two groups receiving either 30 mg/kg CBZ (200 μL) or sterile H_2_O control (200 μL) 5 × weekly.

**Fig. 1:**
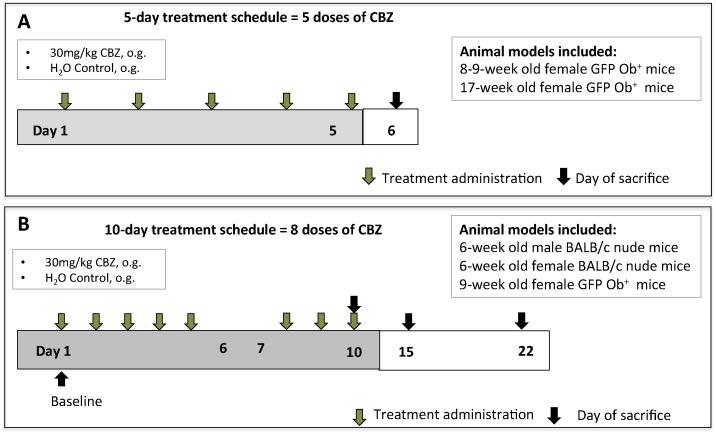
Schematic outline of the *in vivo* studies. (A) 8–9- and 17-week old female GFP Ob^+^ mice (n = 4/group) received 30 mg/kg Cabozantinib (CBZ) or sterile H_2_O control (CTRL) 5 × weekly for 5 days *via* oral gavage. Animals were killed 24 h after the last treatment administration. (B) Male and female 6-week old BALB/c nude as well as 9-week old female GFP Ob^+^ mice (n = 4–5/group) received 8 doses of 30 mg/kg CBZ or sterile H_2_O control (CTRL). Animals were killed 6 h after the last administration. To monitor if treatment effects of CBZ were maintained once treatment was terminated additional female BALB/c nude mice were culled 5 (day 15) and 12 days (day 22) after treatment termination.

To determine short-term effects of CBZ, 8–9- (high bone turnover, n = 4/group) and 17-week old GFP Ob^+^ mice (low bone turnover, n = 4/group) received 30 mg/kg CBZ or sterile H_2_O control 5 × weekly for 5 days (cumulative dose of 150 mg/kg). Animals were killed 24 h after the last treatment (Fig. 1A).

6-week old male and female BALB/c nude (n = 4–5/group) as well as female 9-week old GFP Ob^+^ mice (n = 4/group) were administered 30 mg/kg CBZ or sterile H_2_O control 5 × weekly for 10 days (8 administrations in total, cumulative dose of 240 mg/kg CBZ). To monitor the rate of bone formation Calcein (30 mg/kg, 100 μL, i.p., Sigma-Aldrich) was injected 6 and 2 days pre cull. Additional animals were killed on day 15 and 22 to assess the reversibility of effects 5 and 12 days after the last administration of CBZ, respectively (Fig. 1B).

### 2.4 Sample collection and processing

Immediately following cull, hind legs were collected, fixed in 4% PFA for 72 h. μCT analysis was performed within 72 h, followed by decalcification in 0.5 M EDTA, 0.5% PFA, PBS, pH 8. Whole blood was collected by cardiac puncture and spun down at 4000 rpm for 10 min at 4 °C and serum stored at − 80 °C prior to analysis of bone turnover markers.

### 2.5 Effects of longer-term Cabozantinib treatment

Contralateral, non-tumour bearing tibiae from castrated adult male NOD/SCID mice (injected intratibially with VCaP BM1/cr-luc prostate cancer cells, 30 mg/kg CBZ daily for 15 days, n = 6–7/group) [5], as well as tibiae from 6-week old castrated male beige SCID mice (injected intratibially with C4-2B prostate cancer cells, 60 mg/kg CBZ 5 × weekly for 6-weeks, n = 9–10/group) [32] were analysed to determine long-term treatment effects of CBZ on growth plate cartilage.

### 2.6 Micro computed tomography imaging and analysis of bone integrity

Analysis of bone volume and structure was performed by micro computed tomography imaging of proximal tibiae using a SkyScan 1172 X-ray computed tomography (SkyScan) as reported previously

[35]. Trabecular bone volume (BV/TV in %, the percentage of the volume of interest occupied by binarised solid objects), number (Tb.N. in mm^− 1^) and thickness (Tb.Th. in mm) were determined.

### 2.7 Histological analysis

All histological analysis was performed on 2 non-serial paraffin embedded histological sections (3 μm) using OsteoMeasure software (Osteometrics) and a Leica RMRB upright microscope.

#### 2.7.1 Staining of growth plate cartilage and epiphyseal growth plate analysis

The cationic dyes Safranin-O and toluidine blue were used to visualize acidic proteoglycan in growth plate cartilage of tibiae. Staining was performed according to standard operating procedures.

Area of resting/proliferating and hypertrophic chondrocyte zone were determined using OsteoMeasure software and normalised to total epiphyseal growth plate area by interactively drawing around the corresponding chondrocyte stacks (Fig. 4A).

#### 2.7.2 Bone cell quantification

**Fig. 2:**
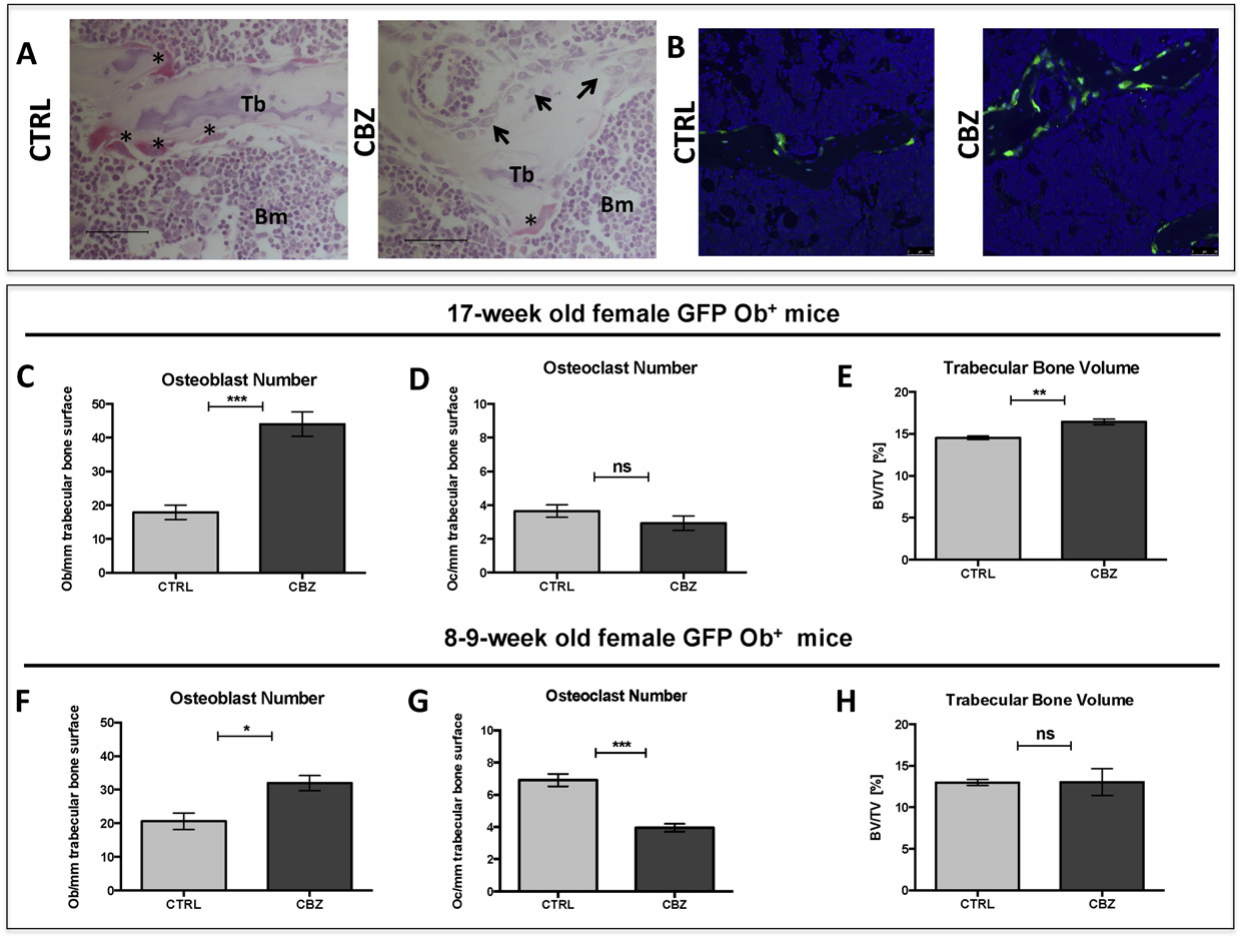
Effects of 5-day treatment with Cabozantinib on bone. (A) Representative TRAP-stained histological bone tissue sections from 8–9 week old mice illustrating the increase in osteoblast and decrease in osteoclast numbers following CBZ treatment *vs.* CTRL. Representative osteoclasts are highlighted with black asterisk, osteoblasts with black arrowhead, 40 × objective, scale bar is 50 μm, BM = Bone marrow, Tb = Trabecular bone. (B) GFP + osteoblastic cells lining trabecular bone surfaces by immunofluorescence, 20 × objective. GFP + osteoblasts are shown in green. (C&F) Osteoblast number and (D&G) osteoclast number/mm trabecular bone surface were scored on H&E- and TRAP-stained tissue sections, respectively after 5-day administration of Cabozantinib (CBZ, 30 mg/kg) or H_2_O control (CTRL). (H&H) Trabecular bone volume (%) of the proximal tibiae determined using μCT analysis. (C–E) represent results for 17-week old (n = 4/group) and (F–H) for 8–9-week old GFP Ob^+^ mice (n = 4/group). Student's t-test: ns is non-significant,* is p ≤ 0.05, ** is p ≤ 0.01, *** is p ≤ 0.001. All data show mean ± SEM.

Tartrate-resistant acid phosphatase (TRAP) staining was used to quantify the number of osteoclasts on histological sections of tibiae and osteoblasts were quantified on H&E stained histological sections — staining was performed in line with previously published studies [34], [35]. Osteoclasts were identified by their bright pink appearance after TRAP staining, multiple nuclei and their ruffled boarder (Fig. 2A). Osteoblasts were identified by their characteristic cuboidal morphology (Fig. 2A) and all trabecular bone surfaces 125 μm away from the growth plate were scored to determine bone cell number/mm trabecular bone excluding all cortical bone surfaces.

Megakaryocytes (identified by their characteristic shape and large lobulated nucleus) number/mm^2^ bone surface was counted manually on H&E-stained histological sections (confirmed by histopathologist K.H.). Quantification commenced 125 μm from the growth plate and an area 1250 μm in length was scored (Fig. 5A).

### 2.8 Visualisation of vascular endothelial cells by immunofluorescence

To visualize effects of CBZ on the bone marrow vasculature, immunofluorescence staining against the endothelial cell marker endomucin was performed. Antigen retrieval was performed using Tris-buffer (15 min, 95 °C), unspecific binding blocked by incubation in 5% normal goat serum/3% BSA and primary antibody (Endomucin V.7C7, rat monoclonal, Santa Cruz, sc-65495) incubated at 4 °C over night. This was followed by incubation in secondary antibody (Alexa fluor 555, goat anti-rat Igg, LifeTechnologies, A21434, 1 h) and images acquired using a Leica DMI4000B microscope and LAS AF Lite software (20 × objective) and a Nikon A1 confocal microscope with NIS Elements software (40 × objective).

### 2.9 Bone turnover markers

Rat/Mouse PINP Enzyme immunoassay for N-terminal propeptide and MouseTRAP™ Assay were performed to determine osteoblast and osteoclast activity, respectively. Assays were performed according to manufacturer's instructions (both Immunodiagnostic Systems, UK).

### 2.10 Statistical analysis

All statistical analysis was performed using Prism GraphPad (Version 6.0). The applied statistical analysis is indicated in each figure legend. Analysis was performed using Student's t-test or two-way ANOVA with Bonferroni post-test. p-Values of p ≤ 0.05 were considered significant.

## 3 Results

### 3.1 The bone microenvironment is modified following 5-days of Cabozantinib administration

In this study we aimed to establish whether short-term treatment with CBZ affects the bone microenvironment. We therefore initially administered 30 mg/kg/day CBZ for 5 days (cumulative dose = 150 mg/kg) and assessed osteoblast and osteoclast number per mm trabecular bone surface, as well as bone volume and structure compared to saline control. We used two cohorts of mice; 8–9-week old animals with a high bone turnover and 17-week old animals with a more mature skeleton, allowing us to compare the effects of CBZ in these different bone microenvironments (Fig. 1).

#### 3.1.1 CBZ effects on osteoblasts

Osteoblasts were identified based on their characteristic morphology and quantified on H&E-stained tissue sections (Fig. 2A). In addition, we used genetically engineered mice expressing GFP in cells of the osteoblastic lineage to confirm the effects of CBZ on osteoblasts by immunofluorescence (hereafter called GFP Ob^+^, Fig. 2B). After 5 daily administrations of 30 mg/kg CBZ, there was significantly increased numbers of osteoblasts lining the trabecular bone surfaces in 17-week old GFP Ob^+^ mice (CTRL: 17.92 ± 2.11 *vs.* CBZ: 44.02 ± 3.63, p ≤ 0.001, Fig. 2C). This effect, was also seen in 8–9-week old GFP Ob^+^ mice, although less prominent (CTRL: 20.60 ± 2.46 *vs.* CBZ: 31.96 ± 2.21, p ≤ 0.05, Fig. 2A,B&F). Despite the increase in osteoblast number, there was no significant difference in osteoblast activity (serum PINP levels) between the treatment groups (17-week old GFP Ob^+^: CTRL: 33.63 ± 2.69 ng/mL *vs.* CBZ: 40.05 ± 3.48 ng/mL, 8–9-week old GFP Ob^+^: CTRL: 77.86 ± 5.69 ng/mL *vs.* CBZ: 84.15 ± 10.64 ng/mL; Supplementary Fig. 1 A&C).

#### 3.1.2 Effects on osteoclasts

The number of osteoclast/mm trabecular bone surface was determined on TRAP-stained histological sections. Five daily administrations of CBZ induced a substantial reduction in osteoclast number/mm trabecular bone surfaces in 8–9-week old GFP Ob^+^ mice compared to CTRL (CTRL: 6.91 ± 0.39 *vs.* CBZ: 3.95 ± 0.24, p ≤ 0.001, Fig. 2A&G). In contrast, osteoclast number was not altered in older (17-week old) GFP Ob^+^ mice (CTRL: 3.65 ± 0.37 *vs.* CBZ: 2.93 ± 0.43, Fig. 2D), probably reflecting the age-related reduction in bone remodelling. Osteoclast activity (TRAP) was not affected by CBZ administration in either model (8–9-week old GFP Ob^+^: CTRL: 8.33 ± 0.69 U/L *vs.* CBZ: 10.12 ± 1.11 U/L; 17-week old GFP Ob^+^: CTRL: 11.36 ± 1.63 U/L *vs.* CBZ: 15.25 ± 1.47 U/L, Supplementary Fig. S1B&D).

#### 3.1.3 Effects on trabecular bone volume and structure

To determine if the effects on osteoblasts and osteoclasts resulted in alterations of bone structure and volume, we performed μCT analysis of proximal tibiae after administration of 30 mg/kg CBZ or CTRL for 5-days. There was significantly increased trabecular bone volume in 17-week old GFP Ob^+^ mice (p ≤ 0.01, Fig. 2E, Table 1), whereas all other analysed bone parameters, including trabecular thickness and number remained unaffected. No CBZ-induced alterations in bone structure were determined in 8–9-week old animals (Fig. 2H, Table 1).

Taken together, these data demonstrate that a 5-day course of CBZ treatment is sufficient to induce modifications of osteoblasts and osteoclasts, although longer-term administration of CBZ might be required to cause significant alterations to bone structure.

### 3.2 A 10-day course of Cabozantinib alters bone structure and modifies key bone cells

As the 5-day administration of 30 mg/kg CBZ (cumulative dose: 150 mg/kg) was found to rapidly modify osteoblast and osteoclast number, with only modest effects on bone volume, we next increased the dosing regimen to a total of 8 administrations of CBZ over 10 days (cumulative dose: 240 mg/kg) (Fig. 1B).

#### 3.2.1 Effects on osteoblasts

**Fig. 3:**
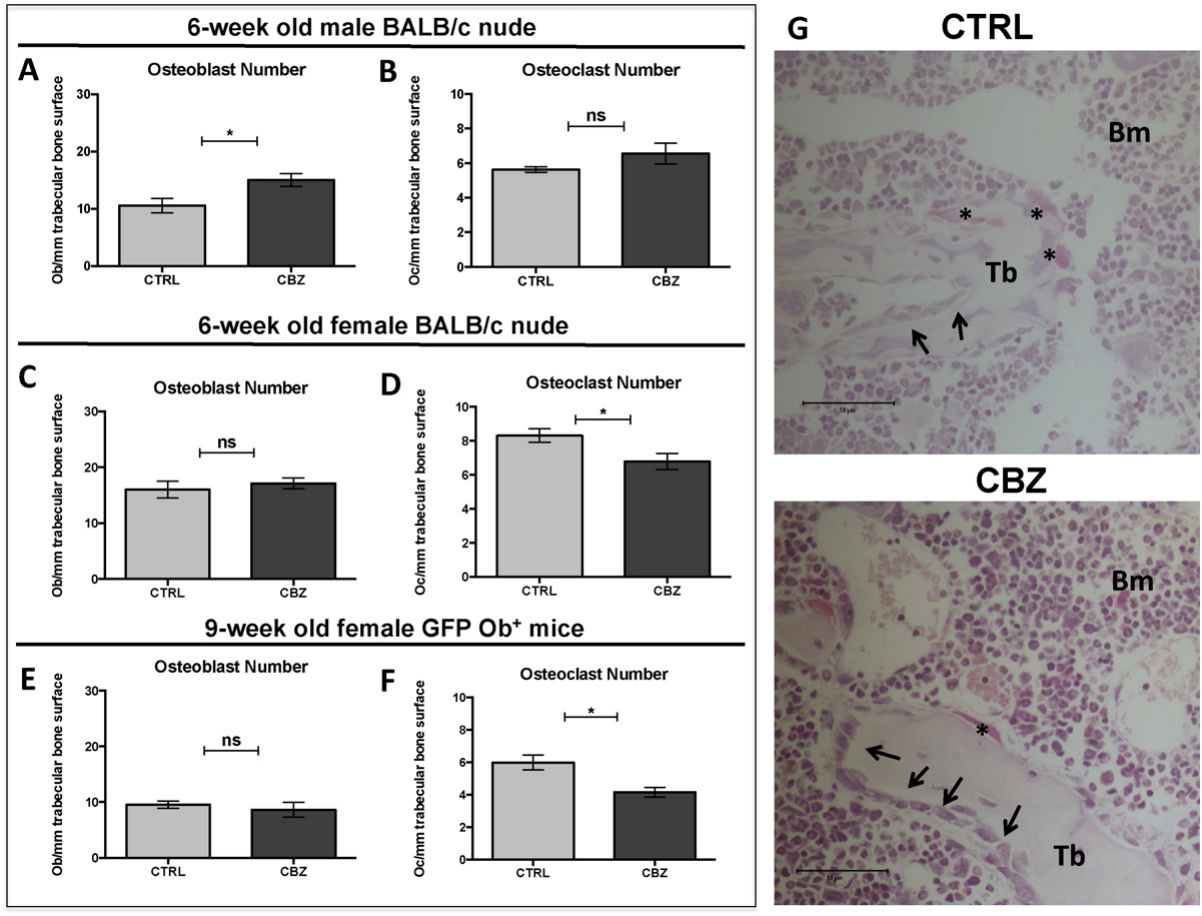
Effects of Cabozantinib on osteoclasts and osteoblasts. (A) Osteoblast number and (B) osteoclast number/mm trabecular bone surface were determined on H&E- and TRAP-stained histological sections after 8 doses of 30 mg/kg CBZ or sterile H_2_O control (CTRL) in 6-week old male BALB/c nude mice (n = 4/group). 6-week old female BALB/c nude (n = 5/group) and 9-week old female GFP Ob + mice (n = 4/group) received the same treatment schedule prior to analysis of (C&E) osteoblast and (D&F) osteoclast number/mm trabecular bone surface. Representative TRAP stained histological sections of 6-week old female BALB/c nude mice are shown in (G). Black asterisk highlights osteoclasts, black arrowheads indicate osteoblasts, 40 × objective, scale bar is 50 μm. Student's t-test: ns is non-significant, * is p ≤ 0.05. All data show mean ± SEM.

We found that 8 doses of CBZ resulted in significantly increased osteoblast number/mm trabecular bone surface in 6-week old male BALB/c nude mice, compared to control (CTRL: 10.54 ± 1.23 *vs.* CBZ: 15.01 ± 1.12, p ≤ 0.05, Fig. 3A). However, there was no effect on osteoblast number in the other experimental animal models receiving this treatment regimen (6-week old female BALB/c nude: CTRL: 16.01 ± 1.48 *vs.* CBZ: 17.12 ± 0.97, Fig. 3C; 9-week old GFP Ob^+^: CTRL: 9.53 ± 0.66 *vs.* CBZ: 8.64 ± 1.33, Fig. 3E). Similar to 5 doses of CBZ, administration of 8 doses did not alter serum PINP levels in these animal models (Supplementary Fig. S1E–G). In addition, no change in osteoblast number or activity between the treatment groups was detected at day 5 and 12 after treatment termination (Day 15 and 22, respectively, data not shown).

#### 3.2.2 Effects on osteoclasts

When compared to control, 8 doses of CBZ did not alter osteoclast number of 6-week old male BALB/c nude mice (CTRL: 5.63 ± 0.16 *vs.* CBZ: 6.56 ± 0.59, Fig. 3B) but resulted in significantly increased osteoclast size (CTRL: 7.10 × 10^− 5^ ± 4.74 × 10^− 6^ mm^2^ *vs.* CBZ: 9.72 × 10^− 5^ ± 6.53 × 10^− 6^ mm^2^, p ≤ 0.05 — data not shown), which may indicate a loss of activity. In contrast, there was a significant decrease in osteoclast number both in 6-week old female BALB/c nude (CTRL: 8.29 ± 0.39 *vs.* CBZ: 6.78 ± 0.48, p ≤ 0.05, Fig. 3D&G) and 9-week old GFP Ob^+^ (CTRL: 5.99 ± 0.46 *vs.* CBZ: 4.15 ± 0.30, p ≤ 0.05, Fig. 3F) treated with CBZ. Osteoclast activity was not significantly altered in 6-week old female BALB/c nude mice (CTRL: 16.21 ± 0.71 U/L *vs.* CBZ: 14.11 ± 2.88 U/L, Supplementary Fig. 1I) and 9-week old GFP Ob^+^ mice (CTRL: 12.04 ± 1.19 *vs.* CBZ: 13.35 ± 1.22 U/L, Supplementary Fig. S1J). However, there was a trend towards increased osteoclast activity in 6-week old male BALB/c nude mice receiving CBZ (CTRL: 14.37 ± 1.05 *vs.* CBZ: 18.85 ± 1.87, p = 0.0817, Supplementary Fig. S1H).

Although the CBZ induced effects on osteoclast number were rapidly reversed after treatment termination (Day 10: CTRL: 8.29 ± 0.39 *vs.* CBZ: 6.78 ± 0.48, p ≤ 0.05; Day 15: 7.55 ± 0.45 *vs.* CBZ: 7.80 ± 0.23; Day 22: CTRL: 9.84 ± 0.35 *vs.* CBZ: 9.99 ± 0.60) osteoclast activity remained significantly elevated compared to control 12 days after administration of the last dose of CBZ (CTRL: 9.99 ± 0.82 U/L *vs.* CBZ: 16.79 ± 2.85 U/L, p ≤ 0.05, Supplementary Fig. S2C).

#### 3.2.3 Effects on trabecular bone volume and structure

We next assessed if 8 doses of CBZ (cumulative dose: 240 mg/kg) caused alterations in trabecular bone volume, number and/or thickness of proximal tibiae. CBZ caused significantly increased trabecular thickness in all the animal models (p ≤ 0.01, Table 1) when compared to control. 9-week old GFP Ob^+^ mice treated with CBZ additionally had increased trabecular number (p ≤ 0.05, Table 1) and volume (p ≤ 0.01, Table 1). These parameters were not significantly affected in BALB/c nude mice (Table 1) although there was a trend towards increased trabecular bone volume in 6-week old male BALB/c nude mice (p = 0.0694 *vs.* CTRL, Table 1). The CBZ-induced increase in trabecular thickness was transient and normalised to control levels 5 days after termination of treatment (Day 15) (Table 1).

These data support that bone effects depend on continuous administration of CBZ and vary according to age (and therefore bone turnover) and sex of the experimental animal, as well as the parameter measured.

### 3.3 Effects of Cabozantinib on the epiphyseal growth plate

**Fig. 4:**
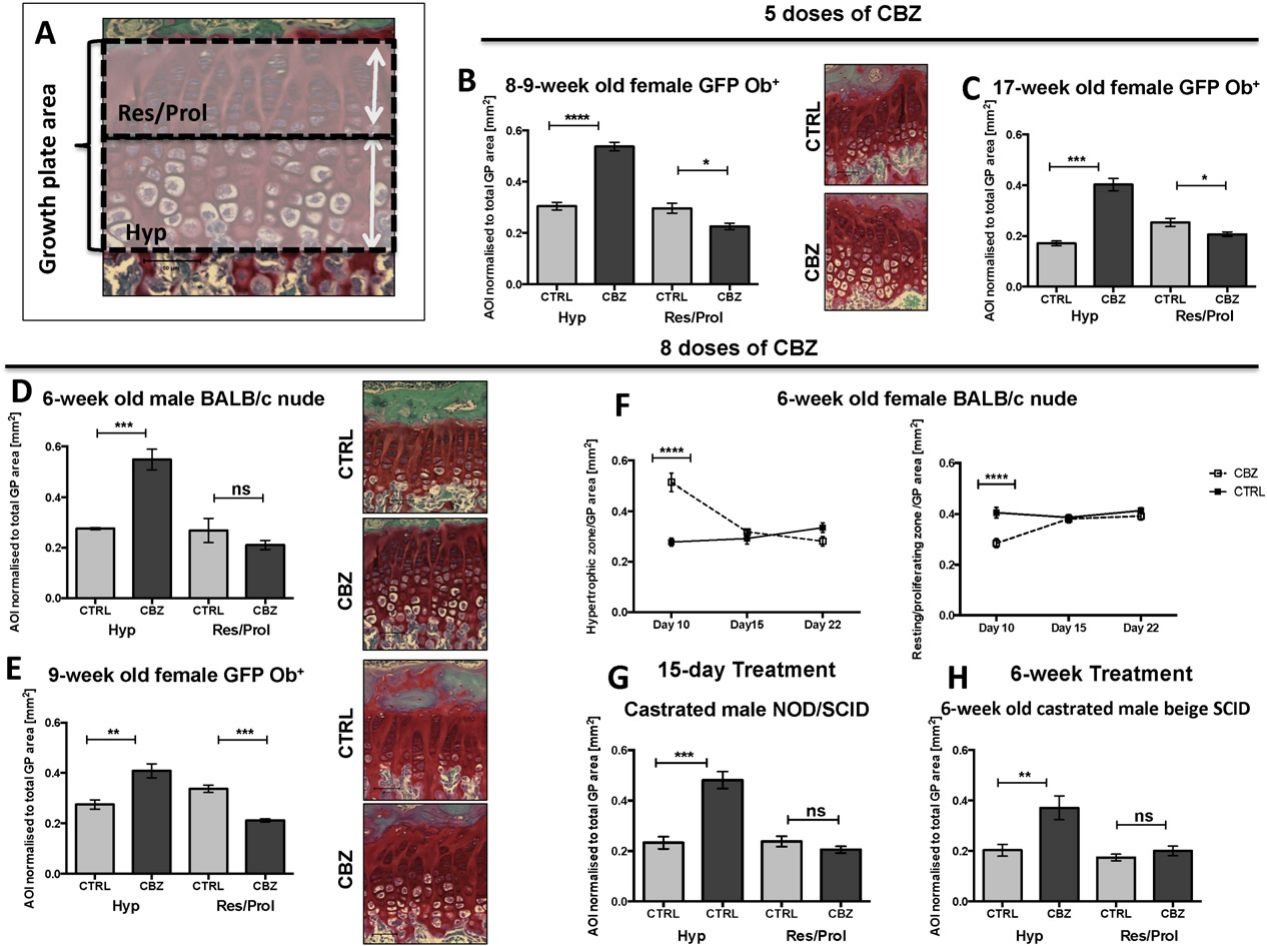
Effects of Cabozantinib on the epiphyseal growth plate. (A) Schematic illustration of growth plate quantification after administration of 30 mg/kg Cabozantinib (CBZ) or sterile H_2_O control (CTRL). It was interactively drawn around the hypertrophic (Hyp) and resting/proliferating (Res/Prol) chondrocyte zone of the epiphysis using OsteoMeasure software. Effects of 5 doses (n = 4/group) of CBZ or CTRL on the epiphyseal growth plate are shown in (B) for 8–9- and (C) 17-week old female GFP Ob^+^ mice. Alterations in growth plate composition following 8 doses of CBZ or CTRL are presented in (D) 6-week old male BALB/c nude (n = 4/group), (E) 9-week old GFP Ob^+^ (n = 4/group) and (F) 6-week old female BALB/c nude mice (day 10: n = 4 CBZ; n = 5/group/time point). Representative Safranin-O stained histological sections of tibiae are shown in (B,D&E). Effects on the growth plate after 30 mg/kg CBZ or CTRL daily for 15 days in NOD/SCID mice (n = 6 CTRL, n = 7 CBZ), are presented in (D). (E) highlights effects after 60 mg/kg CBZ or CTRL 5 × weekly for 6 weeks in 6-week old of castrated male beige SCID mice (n = 9 CTRL, n = 10 CBZ). 20 × objective, scale bar is 50 μm. Student's t-test or (F) two-way ANOVA with Bonferroni post-test, * is p ≤ 0.05, ** is p ≤ 0.01, *** is p ≤ 0.001, **** is p ≤ 0.0001. AOI is area of interest. All data show mean ± SEM.

The epiphyseal growth plate is comprised of cartilage producing chondrocytes embedded in a proteoglycan-rich extra cellular matrix. We noted significant modifications of the epiphysis in CBZ treated animals and therefore quantified the area of resting/proliferating and hypertrophic chondrocytes (in mm^2^), respectively (Fig. 4A) after a 5- and 8-dose regimen of CBZ or CTRL. Additionally we analysed tumour-free tibiae collected in previously published tumour models [5], [32], in order to assess effects of longer-term CBZ treatment.

Five daily administrations of CBZ resulted in a significant increase in the hypertrophic chondrocyte zone in both, 8–9- (p ≤ 0.0001, Fig. 4B, Table 2) and 17-week old female GFP Ob^+^ mice (p ≤ 0.001, Fig. 4C, Table 2) Similarly, a 10-day course (8 administrations) of CBZ increased the hypertrophic chondrocyte area in all the animal models when compared to control (6-week old male BALB/c nude: p ≤ 0.001, Fig. 4D; 9-week old GFP Ob^+^: p ≤ 0.01, Fig. 4E; 6-week old female BALB/c nude: p ≤ 0.0001, Fig. 4F; Table 2). Next to the elongated hypertrophic zones, the chondrocyte stacks appeared disorganized in CBZ treated animals compared to control (Fig. 4B,D&E). The CBZ-induced increase in growth plate thickness was reversed to control levels within 5 days of treatment termination (Fig. 4 F, Table 2). In addition to the increased hypertrophic chondrocyte area the area of resting/proliferating chondrocytes was smaller in animals receiving 5 (8–9-week old GFP Ob^+^: p ≤ 0.05, Fig. 4B; 17-week old female GFP Ob^+^: p ≤ 0.05, Fig. 4C; Table 2) and 10 doses of CBZ (9-week old GFP Ob^+^: p ≤ 0.001, Fig. 4E, Table 2; 6-week old female BALB/c nude: p ≤ 0.0001, Fig. 4F; Table 2). Treatment follow-up analysis in 6-week old female BALB/c nude mice revealed that also the resting/proliferating chondrocyte area reached control levels as quickly as 5 days post CBZ treatment termination (Fig. 4F, Table 2). These results demonstrate that CBZ induces reversible alterations to the epiphyseal growth plate by disrupting chondrocyte differentiation. Material from previously published studies allowed us to determine the effects on the growth plate on histological samples from contralateral, tumour free tibiae following longer-term treatment with CBZ, Table 2 [5], [32]. In agreement with our short-term studies, castrated male NOD/SCID mice that had received daily administration of 30 mg/kg CBZ for 15 days had a significantly elongated hypertrophic chondrocyte area when compared to control mice (p ≤ 0.001, Fig. 4G, Table 2). Similar results were observed after 6-week treatment with 60 mg/kg CBZ (p ≤ 0.01, Fig. 4H) of castrated male beige SCID mice and no alteration in the proliferative/resting chondrocyte zone was determined in either of these experiments (Fig. 4G&H, Table 2).

### 3.4 Effects of Cabozantinib on bone marrow composition

**Fig. 5:**
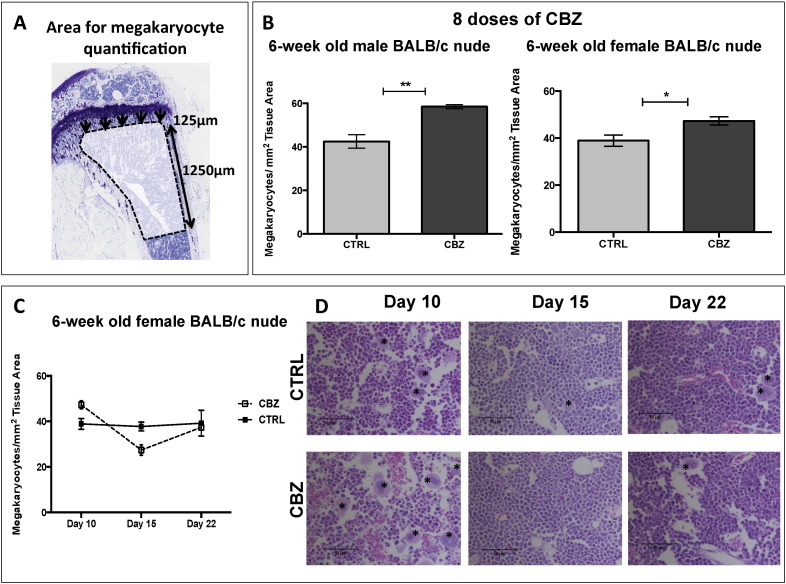
Effects of Cabozantinib on bone marrow cellularity. (A) Megakaryocytes/mm^2^ tissue area were scored 125 μm away from the growth plate as illustrated. An area with a total length of 1250 μm was scored. (B) Number of megakaryocytes was scored after 8 administrations of 30 mg/kg CBZ or sterile H_2_O control for 10 days using 6-week old male (n = 4/group) and female (n = 4-5/group) BALB/c nude mice. (C) Megakaryocyte number 5 (day 15) and 12 days (day 22) after the last CBZ administration in 6-week old female BALB/c nude mice. Representative H&E stained tissue sections illustrating effects on megakaryocytes and bone marrow cellularity are demonstrated in (D), 20 × objective, scale bar is 50 μm. Black asterisk points out megakaryocytes. (B) Student's t-test, * is p ≤ 0.05, ** is p ≤ 0.01. (C) Two-way ANOVA with Bonferroni post-test. All data show mean ± SEM.

During our analyses we observed that CBZ caused alterations in bone marrow composition. In particular, CBZ-treated animals appeared to have increased numbers of red blood cells in the bone marrow associated with numerous megakaryocytes. We therefore determined the effects of CBZ on the number of megakaryocytes/mm^2^ bone tissue by scoring H&E stained sections of tibiae as illustrated in Fig. 5A. In BALB/c nude mice, 8 administrations of CBZ resulted in increased numbers of megakaryocytes/mm^2^ tissue area when compared to control (Male BALB/c nude: CTRL: 42.50 ± 3.09 *vs.* CBZ: 58.52 ± 0.88, p ≤ 0.01, Fig. 5B; Female BALB/c nude: CTRL: 38.91 ± 2.37 *vs.* CBZ: 47.30 ± 1.77, p ≤ 0.05, Fig. 5B&D). Megakaryocyte numbers reduced 5 days after the last administration of CBZ (CTRL: 37.76 ± 1.95 *vs.* CBZ: 27.43 ± 2.30, Fig. 5C) and normalised to control levels by day 12 (CTRL: 39.25 ± 5.66 *vs.* CBZ: 37.44 ± 1.24, Fig. 5C).

**Fig. 6:**
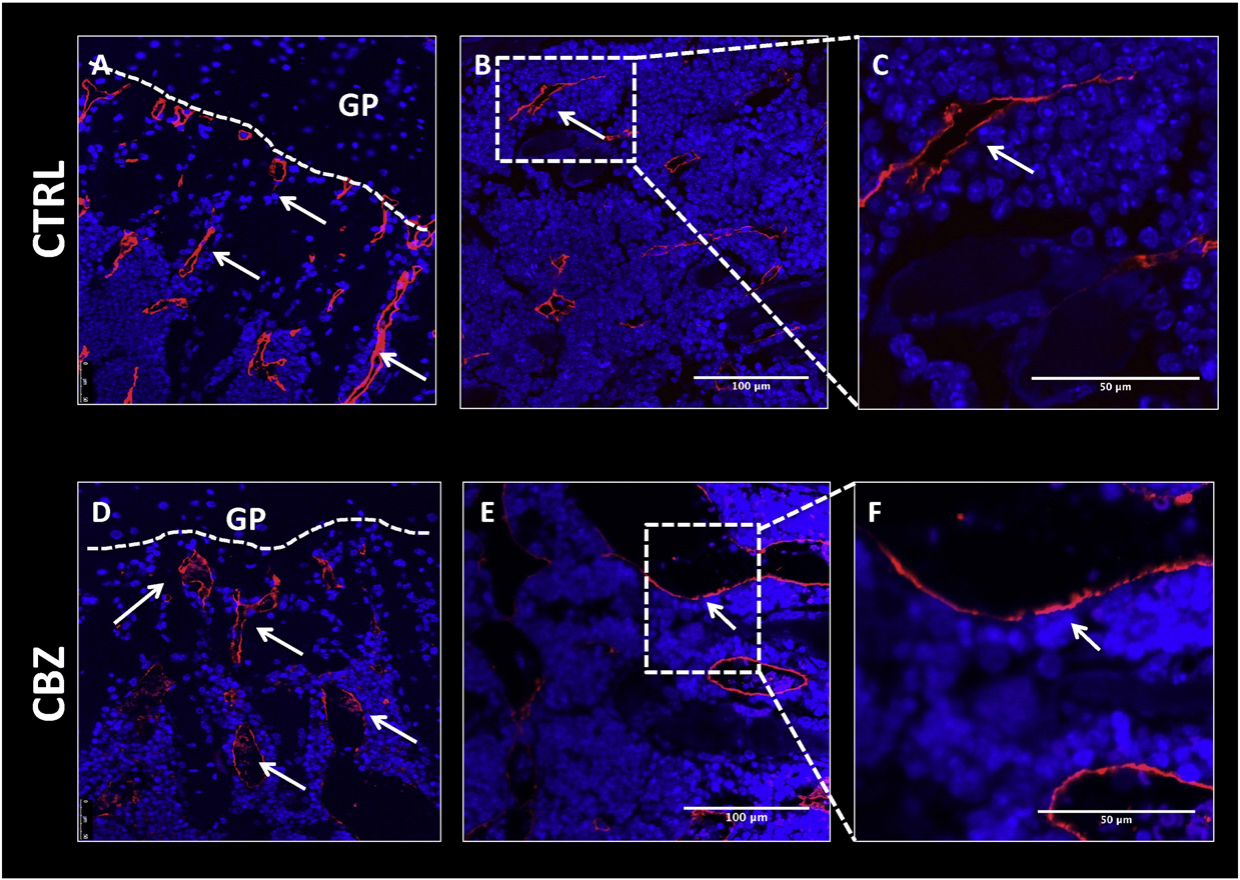
Effects of Cabozantinib on the bone marrow vasculature. Bone marrow vasculature was visualized using immunofluorescence staining against endomucin. Representative histological sections of tibiae from 6-week old male BALB/c nude mice receiving 8 doses 30 mg/kg CBZ (D–F) or sterile H_2_O control (A–C) are shown. Representative images showing vascular composition in the epiphyseal area of the tibia are shown in (A) for control and (D) for CBZ treated mice. 20 × objective, scale bar is 50 μm. CBZ induced effects on bone marrow vascularity (shown in B,C,E &F), 40 × objective and scale bar is 50–100 μm, respectively. GP = growth plate, white arrowheads point out a subset of vessels.

In addition to the changes to the bone microenvironment described above, histological analysis revealed that overall bone marrow cellularity was notably reduced in CBZ treated animals compared to control. Administration of 8 doses of CBZ resulted in vascular ectasia and spillage of mature (non-nucleated) red blood cells amongst the extra vascular bone marrow cells (Fig. 5D). Haemangioma-like bone marrow blood vessels, which were densely filled with erythrocytes, were observed, locally replacing the normal haematopoietic bone marrow (Fig. 5C, Fig. 6D–F). The dilated vessels appeared thin walled compared to the ones observed in control bone marrow although this requires confirmation (Fig. 6A–F). The effects were most prominent after 8 administrations of CBZ, with similar but less prominent alterations observed after 5 doses. Alterations in bone marrow vasculature and spillage of erythrocytes were rapidly lost once CBZ treatment was terminated, with normal bone marrow restored by day 15 (Fig. 5C&D).

Taken together, these data show that CBZ affects the bone microenvironment through modification of multiple cell types, including bone cells and cells of the haematopoietic marrow.

## 4 Discussion

Improving outcome for patients with skeletal metastases requires effective therapeutic targeting of both tumour cells and the supporting bone microenvironment, ideally using a single agent that modifies multiple targets involved in driving both tumour growth and cancer-induced bone disease. The small molecule tyrosine kinase (RTK) inhibitor Cabozantinib (CBZ) is an agent that fits this profile, as it inhibits multiple RTKs that have important roles in tumour growth as well as in bone biology. CBZ has shown activity in clinical trials in prostate cancer, improving progression free survival, reducing pain and resulting in partial and or complete resolution in bone scans [7]. The authors of the study highlighted that CBZ may not only exert effects on the tumour directly, but also indirectly through targeting cells of the bone microenvironment. However, it remains to be established whether these effects are tumour-independent. To date, the majority of experiments investigating CBZ effects on tumour growth in bone have been performed in models of advanced prostate cancer [5], [32], [33], where substantial bone loss and large tumour burden has hampered analysis of CBZ responses on bone. Here we present the first detailed characterisation of the effects of CBZ on tumour-free bone *in vivo*, providing evidence that the treatment effects of CBZ might be partially mediated by cells of the bone microenvironment. Breast cancer preferentially metastasises to bone hence indicating the potential of CBZ as a potential treatment. We therefore included female animal models in our experiments.

Osteoblasts and osteoclasts are key players in the vicious cycle driving progression of bone metastasis [36], and crucially both cell types express receptors targeted by CBZ [2], [3], [4]. In agreement with this, CBZ modifies proliferation and differentiation of both osteoclasts and osteoblasts *in vitro*. A recent study reported inhibition of differentiation and resorptive activity in RAW pre-osteoclast cells in addition to reduced viability and osteocalcin levels (a marker for late osteoblast differentiation) in pre-osteoblastic MC3T3-E1 and mouse bone marrow stromal ST-2 cell cultures following CBZ treatment (0.01–5 μmol/L) [33]. CBZ altered alkaline phosphatase activity (a marker for early osteoblast differentiation) in a biphasic fashion but did not modify mineralisation [33]. In contrast, Nguyen et al. found an increase in mineralisation, reduced proliferation and stimulated alkaline phosphatase activity in MC3T3 cells as a consequence of CBZ treatment (0.01–3 μmol) [32]. Stern and colleagues also reported that treatment of osteoblastic MC3T3-E1 cells with 3 μM CBZ for 24 h reduced the expression of RANKL and alkaline phosphatase and inhibited proliferation in a dose dependent fashion after 48 h [37]. A 3 μM dose of CBZ did not alter expression of TRAP and cathepsin K in RAW 264.7 pre-osteoclastic cells. However, co-treatment with 2–3 μM CBZ and 20 ng/mL RANKL for 5 days (to promote osteoclastogenesis) inhibited the expected RANKL-induced increase in TRAP activity and cell proliferation [37]. The authors concluded that CBZ may act on bone through more than one mechanism, based on the biphasic effects caused by different concentrations of CBZ. In addition, Schimmoller and colleagues have reported a dose-dependent decrease of osteoclast differentiation by CBZ *in vitro* that did not impede the ability of mature osteoclasts to resorb bone. They also found a biphasic effect of CBZ, with increased osteoblast differentiation and bone forming activity at the lower doses but a reduction at higher doses [38]. *In vivo* the tight coupling between osteoblasts and osteoclasts makes it difficult to separate the direct/indirect effects of therapeutic agents on these cells, in particular when both cell types express the target receptor(s). It is possible that CBZ reduced RANKL expression by the osteoblasts in our study, resulting in decreased osteoclast number. However it is not possible to accurately measure the levels of active RANKL in the bone microenvironment and the role of soluble RANKL in regulating bone turnover is unclear. Although we were unable to detect a CBZ-mediated reduction in osteoclast (serum TRAP) activity at the end of the study, the highly significant increase in the length of the hypertrophic chondrocyte zone provides strong evidence that resorption is impaired.

The *in vivo* effects of CBZ on osteoblasts and osteoclasts in the absence of tumour cells have not been investigated in detail. We therefore used a variety of *in vivo* models, including mice of different ages, sex and strain, to perform the first comprehensive analysis of the effects of CBZ on osteoblast and osteoclast number in a range of bone microenvironments. Here we report that CBZ is able to modify bone cells in the absence of tumour. Performing experiments across a spectrum of animal models (young/old, female/male) enabled us to determine the degree of variability between these. Interestingly, we observed inconsistency in the effects CBZ on osteoblasts and osteoclasts depending on the age of the animal model. This demonstrates that the level of bone turnover (decreasing with age) may be important in determining the effects of this agent on bone. Overall, CBZ rapidly reduced the number of osteoclasts/mm trabecular bone surface resulting in increased trabecular thickness after a cumulative dose of 240 mg/kg (8 administrations). Osteoblast numbers/mm trabecular bone was significantly increased after 5 doses of CBZ (150 mg/kg cumulative dose) however no effects on osteoblasts were observed after 8 administrations in female animals (Fig. 3C&E). Male BALB/c nude mice showed a slight increase in osteoblast numbers. It remains unclear why we observe differences in osteoblast and osteoclast number in mice of different sex, however we found that CBZ-induced alterations of megakaryocyte number, bone marrow vasculature, trabecular thickness and elongated hypertrophic chondrocyte zone in the epiphyseal growth plate area were all consistently observed in both male and female mice. Our experiments therefore highlight the need to perform studies in different animal ages/sex in order to demonstrate consistency of therapeutic effects in model systems.

Only limited data are available from tumour models evaluating CBZ-induced effects on bone cells and to our knowledge the majority have been performed in tumour-bearing models. In a study of prostate cancer-induced bone disease, male NOD/SCID mice receiving 30 mg/kg CBZ for 15 days had significantly reduced numbers of osteoclasts/mm trabecular bone surface along the edge of the growth plate of non-tumour bearing tibiae [5]. Nude mice injected intratibially with the prostate cancer cell line ARCaP_M_ receiving 10 or 30 mg/kg CBZ daily for 7 weeks showed increased numbers of osteoblasts but no change in osteoclast numbers in trabecular bone when compared to control [38]. In addition, Dai and colleagues report increased osteoblast perimeter and decreased osteoclast perimeter in non-tumour bearing tibiae of male SCID mice with intratibial PC-3^luc^ prostate tumours, following daily administration of 60 mg/kg CBZ for 3 weeks [33]. Serum PINP and osteocalcin levels were unaffected by CBZ but TRAP5b levels increased. The authors therefore suggest that CBZ might not affect the resorptive activity of osteoclasts but rather inhibit osteoclast maturation [33].

The bone serum marker levels (TRAP and PINP) did not correspond with the observed alterations in bone cell number (Supplementary Fig. S1), due to the design of our experiments. Serum samples for bone marker measurements were only collected at the end of the experiment (reflecting one point in time). In contrast, histological analysis quantifies accumulated effects of CBZ over the entire experimental period. Additionally, serum TRAP/PINP levels represent levels released from the entire skeleton whereas we only quantified bone cell number in tibia, potentially contributing to the observed differences.

Although 5 administrations of CBZ increased osteoblast and decreased osteoclast numbers, this short-term schedule did not result in any significant increase in trabecular bone volume, apart from in 17-week old female GFP Ob^+^ mice (Fig. 2). Increasing the schedule to 8 doses of CBZ over 10 days did result in increased trabecular thickness in all the models evaluated in addition to increased trabecular bone volume and number in 9-week old female GFP Ob^+^ mice (Table 1). This increase was rapidly lost, returning to control levels 5 days after the last administration of CBZ (Table 1). Although the GFP Ob^+^ mice represent a slightly different bone microenvironment compared to BALB/c nude mice, the differential effects of CBZ on trabecular bone is most likely due to the difference in bone turnover between the mice aged 6 and 9 weeks. Our findings highlight that long-term and continuous treatment might be required for bone volume to be altered, but following cessation of treatment the bone microenvironment has the capacity to reverse the alterations. In contrast to our results, Dai and colleagues found no increase in bone mineral content in tumour free murine tibiae receiving 60 mg/kg CBZ for 3 weeks (PC3 tumours) and 5 weeks (Ace1 and C4-2B tumours), respectively. The authors suggested that it may take longer to see treatment effects on bone than the 7-week treatment course [33]. However, 6-week treatment with 60 mg/kg CBZ in castrated and intact mice injected with LuCaP 23.1 prostate cancer cells resulted in significantly increased trabecular bone volume and number in contralateral non-tumour bearing tibiae [32]. These differences may be due to the models and CBZ schedules used, and further studies are required to firmly establish the optimal CBZ dosing regimen required to increase bone volume.

We found substantial alterations in the epiphyseal growth plate of CBZ treated mice, including expansion of the hypertrophic chondrocyte zone. This was prominent in the epiphysis of all animals irrespective of age, sex and treatment schedule. When we analysed growth plates of tumour-free tibiae from animals receiving longer-term CBZ treatment [5], [32] similar effects were observed (Fig. 4). During endochondral ossification, newly synthesized cartilage is replaced by woven bone in a highly organised process involving chondrocyte proliferation, maturation, hypertrophy and finally matrix calcification. There are conflicting views about the terminal fate of the chondrocyte, including their differentiation into osteoblastic cells [39] or apoptosis (reviewed in [40]). It has previously been reported that inhibition of VEGF signalling results in growth plate thickening as a consequence of the expanded hypertrophic chondrocyte zone [4], [41] and this could also be the mechanism involved in the CBZ-induced effects. The growth plate normalised to control thickness 12 days after treatment termination, coinciding with an increase in osteoclast activity (Supplementary Fig. S2). During endochondral ossification, osteoclasts are recruited to the mineralizing front of the growth plate, allowing resorption of cartilage and invasion of osteoblasts to mineralise the matrix, which is a potential explanation for the sudden increase in osteoclast activity once CBZ administration ceased. New blood vessel formation is apparent in the developing epiphysis and anti-VEGF treatment has been shown to modify the growth plate. Children with open growth plates therefore require close monitoring when receiving anti-angiogenic therapy as they are in risk of developing growth plate abnormalities [42]. Our results suggest that this approach is appropriate also for CBZ, although preliminary safety data from a phase 1 trial of CBZ in paediatric cancer patients did not indicate clinical consequences of potential growth plate effects [43].

We also noted alterations in bone marrow composition in CBZ treated animals compared to control, including vascular ectasia and spillage of mature red blood cells in the extra vascular bone marrow (Fig. 6). Dilated blood vessels densely filled with erythrocytes were observed in the bone marrow of mice that had received 8 administrations of CBZ (Fig. 6). Mice that received 15 days of treatment [5] appeared to have reduced haematopoietic cells in the marrow, increased numbers of adipocytes and reduced megakaryocyte cytoplasm, although we were unable to accurately quantify this due to the limited number of samples available from this study. Analysis of histological sections from tibiae of mice receiving 60 mg/kg CBZ 5 × weekly for 6 weeks [32] showed similar changes in bone marrow composition in 3 out of 8 assessed mice, with additional animals displaying a more modest effect. These responses are likely a result of inhibition of VEGFR affecting the bone microvasculature. In support of this, a study using the soluble VEGF receptor chimeric protein Flt(1–3)-IgG reported an increase in thickness of secondary trabeculi *in vivo*. In addition, animals displayed disorganized and dilated blood vessels adjacent to the hypertrophic chondrocyte zone [4] with similar morphology as observed in our studies (Fig. 7A&D).

Next to fatigue, hypertension and hand–foot syndrome were the most common experienced grade 3 adverse events caused by CBZ in patients with advanced prostate cancer [7]; lymphopenia, neutropenia and thrombocytopenia in addition to haemorrhage have been reported as common haematological adverse effects [44]. The bone marrow effects we observed in our experiments may therefore reflect some of the known complications experienced by patients treated with CBZ.

One of our most intriguing findings was that CBZ treatment caused a significant increase in the number of megakaryocytes in the bone marrow (Fig. 5), indicating that CBZ may have impaired terminal differentiation of megakaryocytes and release of platelets. The increase in red blood cells in the marrow could be in agreement with thrombocytopenia reported as a common laboratory abnormality in patients receiving CBZ [44]. However, as with all the CBZ-induced effects detected in our study, megakaryocyte numbers and the associated bone marrow effects rapidly normalised to control levels once treatment was terminated (Fig. 6). Megakaryocytes also play a role in bone homeostasis, including in the maintenance of bone mass [45]. Mice deficient in the transcription factors required for megakaryocyte differentiation and maturation have high numbers of immature megakaryocytes in the bone marrow, accompanied by dramatically increased osteoblast numbers and bone volume [26]. *In vitro*, osteoblast proliferation is increased by the presence of megakaryocytes and direct cell-to cell contact between both cell types is suggested to occur *via* gap junctions [46] and/or integrins [47]. However, to what extent osteoblasts and megakaryocytes are in direct contact *in vivo* remains to be established. In the present study, megakaryocytes did not appear to be in close proximity to osteoblasts in histological sections of tibiae (Fig. 5). Megakaryocytes have also been demonstrated to inhibit formation of osteoclasts when added to pre-osteoclast cultures and to impair osteoclast function [28], [48]. It is therefore possible that CBZ may increase bone volume not only through direct effects on osteoblasts and osteoclasts, but also indirectly through increasing the number of megakaryocytes that in turn stimulate osteoblasts and inhibit osteoclast activity and maturation.

## 5 Conclusion

Collectively our data suggest that CBZ exerts effects on different cell types in the bone microenvironment including cells of the bone marrow. Response of key bone cells to CBZ administration are rapid and reversible once treatment is stopped, supporting the conclusion that continuous administration is required in order to maintain the effects of this agent.

The following are the supplementary data related to this article

Supplementary Fig. S1 Short-term treatment effects of Cabozantinib on osteoclast and osteoblast activity. Serum Tartrate-resistant Alkaline Phosphatase (TRAP) levels as a marker for osteoclast activity and serum Type I procollagen (PINP) levels as a marker for osteoblast activity were measured after 5-day treatment with 30 mg/kg CBZ or sterile H_2_O control for (A–B) 17-week old and (C–D) 8–9 week old GFP Ob^+^ mice (n = 4/group). Bone cell activity determined after 8 doses of CBZ or CTRL is shown in (E&H) for 6-week old male BALB/c nude (n = 4/group), (F&I) 6-week old female BALB/c nude (n = 4–5/group) and (G&J) 9-week old GFP + mice (n = 4/group). Student's t-test: ns is non-significant. All data show mean ± SEM.

Supplementary Fig. S2 Alterations in growth plate structure and osteoclast activity following Cabozantinib treatment termination. 6-week old female BALB/c nude mice received 30 mg/kg CBZ or sterile H_2_O control (CTRL) 5 × weekly 10 days (8 administrations in total). (A) Shows representative Safranin-O stained sections of tibiae illustrating treatment effects of CBZ on the epiphysis after 8 administrations (day 10), 5 and 12 days after treatment has been terminated. Quantitative data are shown in (B) (day 10: n = 4 for CBZ, n = 5 for CTRL; n = 5/group for all other time points). Analysis of osteoclast activity measured by serum TRAP levels are shown in (C) (n = 5/group and time point). *p ≤ 0.05, ****p ≤ 0.0001. Two-way ANOVA with Bonferroni post-test. All data show mean ± SEM.

## Author contributions

MTH performed experiments, participated in study design, carried out analysis of data, data presentation and participated in drafting and revising the manuscript IH participated in study design, data interpretation, critical evaluation, revision and drafting of the manuscript. NJ Brown participated in drafting and revising the manuscript and critically reviewed its content in addition to holding the PPL for the *in vivo* studies and providing advice on *in vivo* protocols and individual study plans. KH assessed histological sections, assisted with data interpretation and manuscript revision. TND generated the pOBCol2.3GFPemd mice. RH participated in experimental optimisation and manuscript revision. SP Robinson, TJ Graham and E Corey provided samples from their related experimental work and reviewed the manuscript. All authors take responsibility for their work, have read and given final approval for publication of this manuscript.

## Conflict of interest

This study was partially funded by a research grant from Exelixis Inc.

## Source of funding

This research was supported by Exelixis, Inc. (R/136892), Breast Cancer Campaign (UK) (2010NovPhD17) and CR-UK Imaging Centre grant (C1060/A10334).

## Competing interests

The authors declare that there are no competing interests.

## Figures and Tables

**Table 1:** Analysis of bone structure and volume. Trabecular bone volume (BV/TV in %), number (in mm^− 1)^ and thickness (in mm) of proximal tibiae were analysed after 5 and 8 dose regimen of 30 mg/kg Cabozantinib (CBZ) or control (CTRL) using μCT analysis. Students t-test or ^(1)^two-way ANOVA with Bonferroni post-test was used for statistical analysis. ns is non-significant, * is p ≤ 0.05, ** is p ≤ 0.001. BV/TV = trabecular bone volume, Tb.N. = trabecular number, Tb.Th. = trabecular thickness.

**Analysis of treatment effects on bone volume and structure**									
	**BV/TV (%)**			**Tb.N. (mm^− 1^)**			**Tb.Th. (mm)**		
	**CTRL**	**CBZ**	**p**	**CTRL**	**CBZ**	**p**	**CTRL**	**CBZ**	**p**
**5-day treatment schedule**									
GFP Ob + mice 8–9-week old	12.97 ± 0.36	13.04 ± 1.60	ns	2.86 ± 0.05	2.77 ± 0.36	ns	0.045 ± 0.001	0.047 ± 0.001	ns
GFP Ob + mice 17-week old	14.53 ± 0.22	16.42 ± 0.35	**	2.57 ± 0.09	2.72 ± 0.05	ns	0.057 ± 0.002	0.060 ± 0.002	ns

**10-day treatment schedule**									
Male BALB/c nude 6-week old	12.16 ± 0.84	16.01 ± 1.53	ns	3.39 ± 0.24	3.84 ± 0.30	ns	0.036 ± 0.001	0.042 ± 0.001	**
Female BALB/c nude 6-week old	11.61 ± 1.31	12.18 ± 1.47	ns ^(1)^	3.21 ± 0.31	2.817 ± 0.303	ns ^(1)^	0.036 ± 0.001	0.043 ± 0.001	** ^(1)^
Female GFP Ob + mice 9-week old	13.48 ± 0.54	18.75 ± 1.09	**	2.65 ± 0.13	3.294 ± 0.146	*	0.051 ± 0.001	0.057 ± 0.001	**

**Follow-up treatment (6-week old female BALB/c nude)**									
5 days post-treatment termination	11.07 ± 1.29	9.86 ± 0.95	ns ^(1)^	2.86 ± 0.33	2.41 ± 0.18	ns ^(1)^	0.039 ± 0.001	0.041 ± 0.001	ns ^(1)^
12 days post-treatment termination	10.49 ± 0.85	11.64 ± 1.49	ns ^(1)^	2.65 ± 0.19	2.83 ± 0.30	ns ^(1)^	0.039 ± 0.001	0.041 ± 0.002	ns ^(1)^

**Table 2:** Treatment effects of Cabozantinib (CBZ) on the hypertrophic and resting/proliferating chondrocyte zone of the epiphyseal growth plate area (in mm^2^) were analysed after 5 and 8 dose regimen of 30 mg/kg CBZ or control (CTRL) on histological sections of tibiae using OsteoMeasure software and normalised to total growth plate area. Student's t-test was used for statistical analysis or ^(1)^two-way ANOVA with Bonferroni post-test. ns is non-significant, * is p ≤ 0.05, ** is p ≤ 0.01, *** is p ≤ 0.001, **** is p ≤ 0.0001.

**Hypertrophic chondrocyte zone area (mm^2^)**			
	**CTRL**	**CBZ**	**p**
**5 doses of CBZ**			
8–9-week old GFP Ob + mice	0.305 ± 0.015	0.537 ± 0.017	****
17-week old GFP Ob + mice	0.171 ± 0.010	0.403 ± 0.025	***

**8 doses of CBZ**			
6-week old male BALB/c nude	0.276 ± 0.004	0.549 ± 0.041	***
6 week old female BALB/c nude	0.278 ± 0.016	0.514 ± 0.035	**** ^(1)^
8–9-week old female GFP Ob^+^	0.275 ± 0.018	0.409 ± 0.028	**

**Treatment follow-up**			
5 days post-treatment termination	0.292 ± 0.022	0.319 ± 0.005	ns ^(1)^
12 days post-treatment termination	0.335 ± 0.020	0.282 ± 0.019	ns ^(1)^

**Longer term treatment**			
Castrated male NOD/SCID mice	0.232 ± 0.024	0.481 ± 0.034	***
Castrated male beige SCID mice	0.203 ± 0.023	0.372 ± 0.047	**

**5 doses of CBZ**			
8–9-week old GFP Ob^+^	0.297 ± 0.019	0.226 ± 0.012	*
17-week old GFP Ob^+^	0.254 ± 0.016	0.207 ± 0.009	*

**8 doses of CBZ**			
6-week old male BALB/c nude	0.269 ± 0.048	0.211 ± 0.018	ns
6 week old female BALB/c nude	0.406 ± 0.021	0.285 ± 0.017	**** ^(1)^
8–9-week old female GFP Ob^+^	0.337 ± 0.014	0.212 ± 0.006	***

**Treatment follow-up (6-week old female BALB/c nude)**			
5 days post-treatment termination	0.387 ± 0.008	0.381 ± 0.016	ns ^(1)^
12 days post-treatment termination	0.414 ± 0.011	0.392 ± 0.014	ns ^(1)^

**Longer term treatment**			
Castrated male NOD/SCID mice	0.238 ± 0.021	0.205 ± 0.013	ns
Castrated male beige SCID mice	0.174 ± 0.013	0.200 ± 0.019	ns
