# Supplementary material for: Rapid modification of the bone microenvironment following short-term treatment with Cabozantinib in vivo
Source: Bone. 2015 Dec;81:581–92. doi: 10.1016/j.bone.2015.08.003 (PMC4768060; doi:10.1016/j.bone.2015.08.003)
Supplement: Supplementary Fig. S1 — Short-term treatment effects of Cabozantinib on osteoclast and osteoblast activity. Serum Tartrate-resistant Alkaline Phosphatase (TRAP) levels as a marker for osteoclast activity and serum Type I procollagen (PINP) levels as a marker for osteoblast activity were measured after 5-day treatment with 30 mg/kg CBZ or sterile H2O control for (A–B) 17-week old and (C–D) 8–9 week old GFP Ob+ mice (n = 4/group). Bone cell activity determined after 8 doses of CBZ or CTRL is shown in (E&H) for 6-week old male BALB/c nude (n = 4/group), (F&I) 6-week old female BALB/c nude (n = 4–5/group) and (G&J) 9-week old GFP + mice (n = 4/group). Student's t-test: ns is non-significant. All data show mean ± SEM. [file mmc1.pdf]

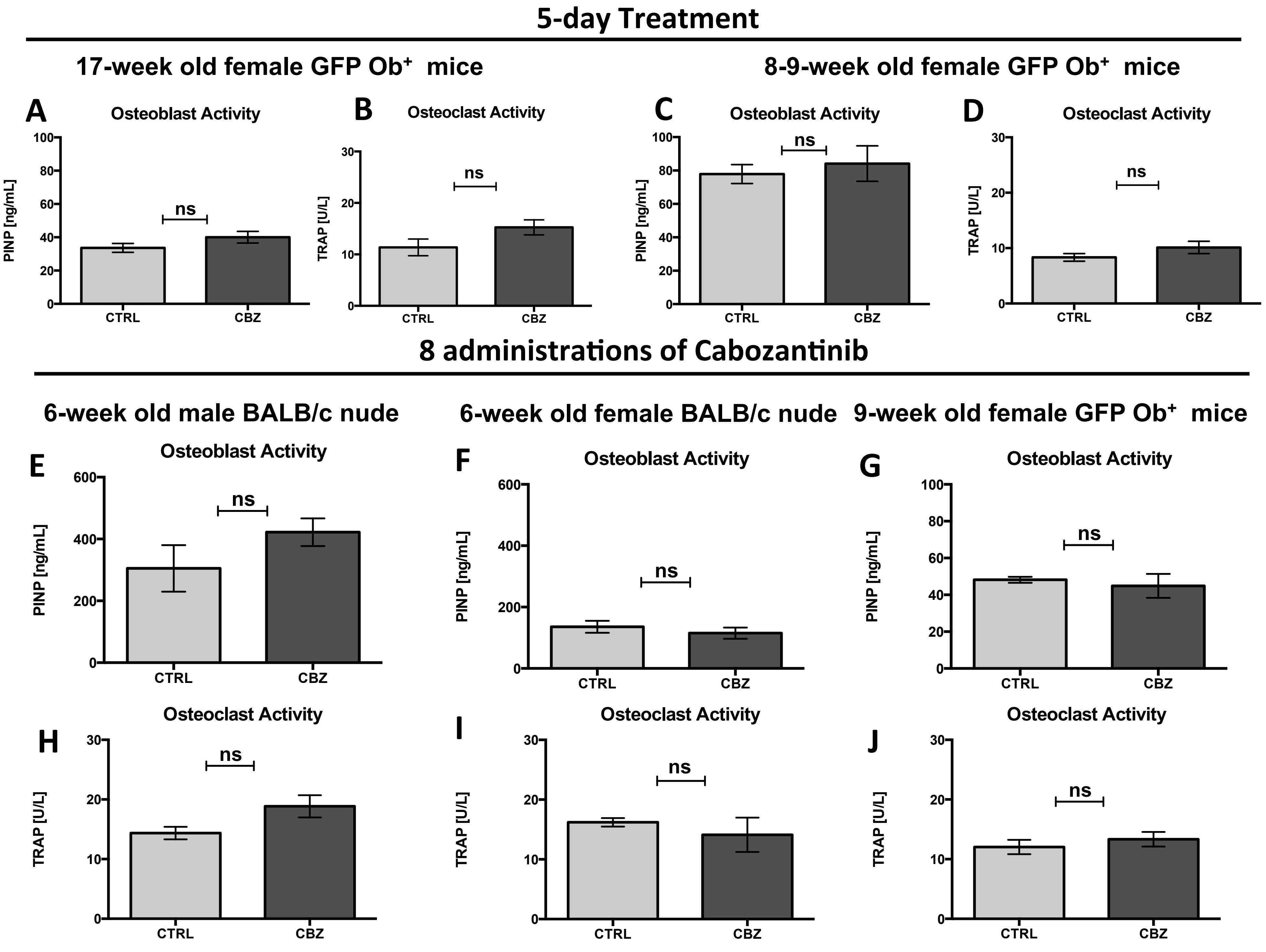
5-day Treatment
17-week old female GFP Ob+ mice
8-9-week old female GFP Ob+ mice
A
Osteoblast Activity
PINP [ng/mL]
100
80
60
40
20
0
ns
CTRL
CBZ
B
Osteoclast Activity
TRAP [U/L]
30
20
10
0
ns
CTRL
CBZ
C
Osteoblast Activity
PINP [ng/mL]
100
80
60
40
20
0
ns
CTRL
CBZ
D
Osteoclast Activity
TRAP [U/L]
30
20
10
0
ns
CTRL
CBZ
8 administrations of Cabozantinib
6-week old male BALB/c nude
6-week old female BALB/c nude
9-week old female GFP Ob+ mice
E
Osteoblast Activity
PINP [ng/mL]
600
400
200
0
ns
CTRL
CBZ
F
Osteoblast Activity
PINP [ng/mL]
600
400
200
0
ns
CTRL
CBZ
G
Osteoblast Activity
PINP [ng/mL]
100
80
60
40
20
0
ns
CTRL
CBZ
H
Osteoclast Activity
TRAP [U/L]
30
20
10
0
ns
CTRL
CBZ
I
Osteoclast Activity
TRAP [U/L]
30
20
10
0
ns
CTRL
CBZ
J
Osteoclast Activity
TRAP [U/L]
30
20
10
0
ns
CTRL
CBZ
