# Supplementary figures and images for: Rapid modification of the bone microenvironment following short-term treatment with Cabozantinib in vivo
Source: Bone. 2015 Dec;81:581–92. doi: 10.1016/j.bone.2015.08.003 (PMC4768060; doi:10.1016/j.bone.2015.08.003)

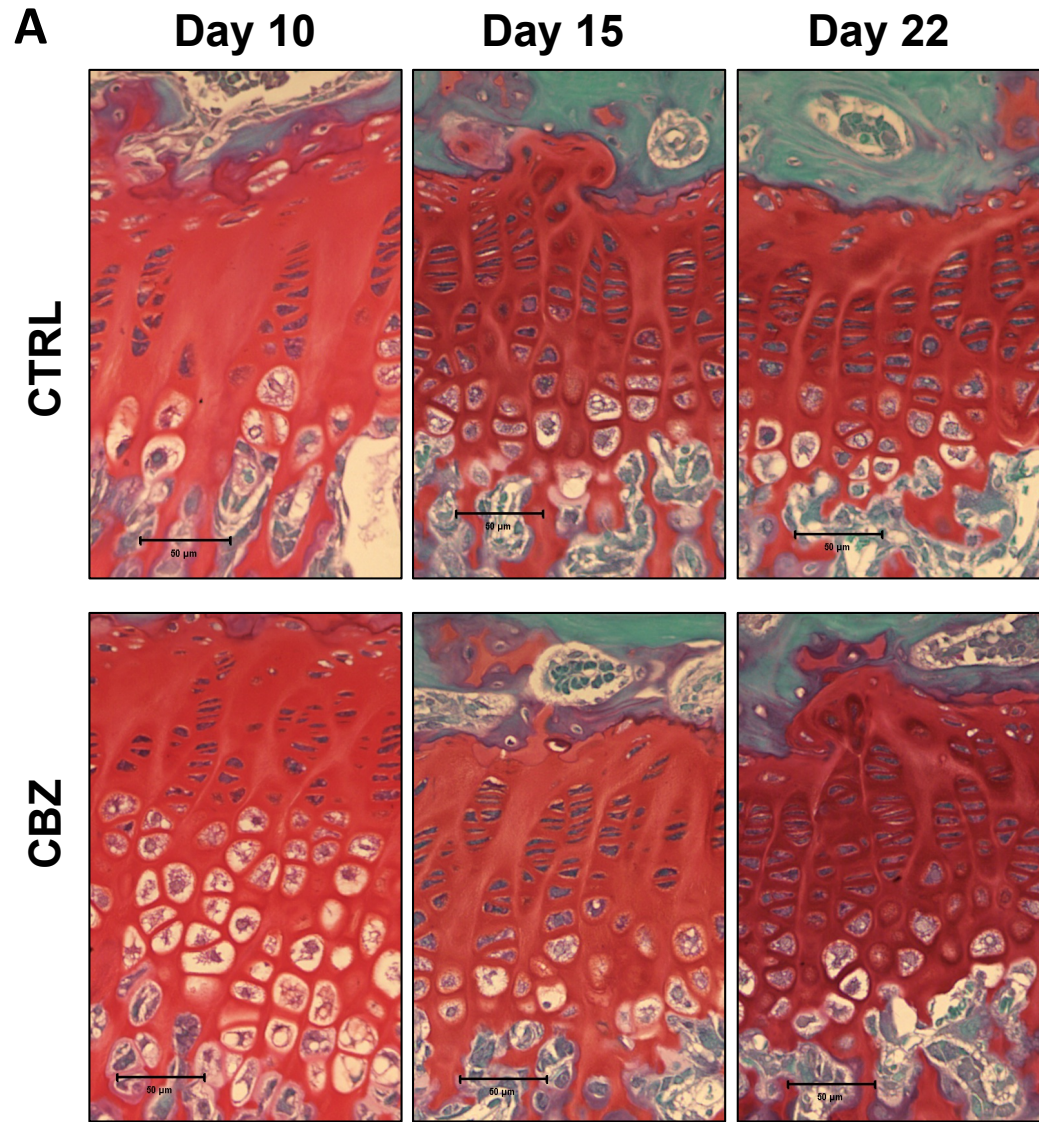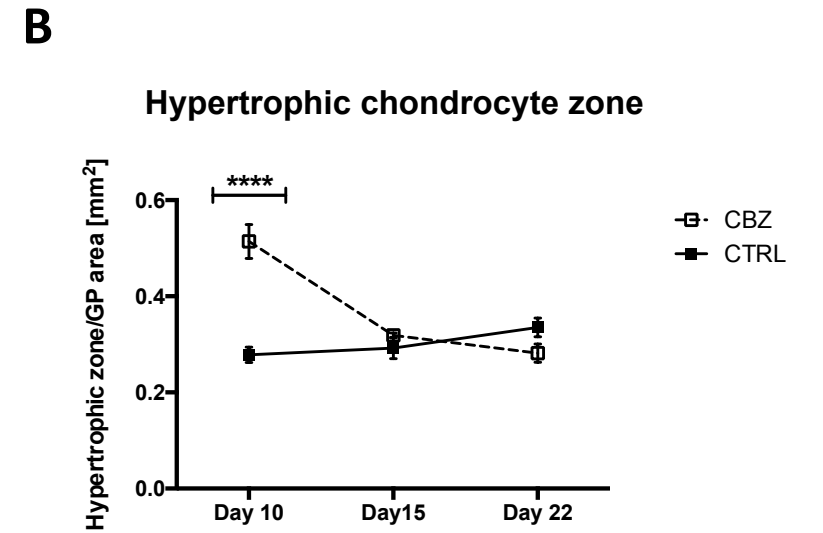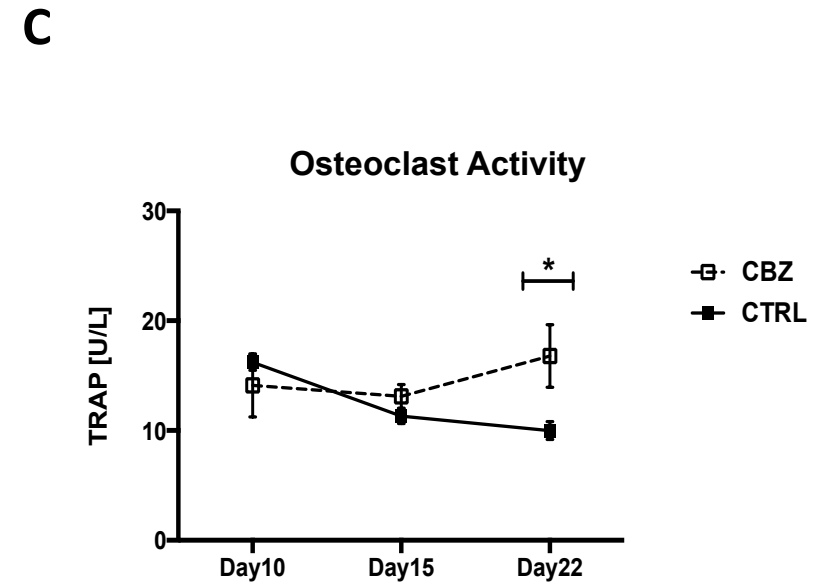

Supplement: Supplementary Fig. S2 — Alterations in growth plate structure and osteoclast activity following Cabozantinib treatment termination. 6-week old female BALB/c nude mice received 30 mg/kg CBZ or sterile H2O control (CTRL) 5 × weekly 10 days (8 administrations in total). (A) Shows representative Safranin-O stained sections of tibiae illustrating treatment effects of CBZ on the epiphysis after 8 administrations (day 10), 5 and 12 days after treatment has been terminated. Quantitative data are shown in (B) (day 10: n = 4 for CBZ, n = 5 for CTRL; n = 5/group for all other time points). Analysis of osteoclast activity measured by serum TRAP levels are shown in (C) (n = 5/group and time point). *p ≤ 0.05, ****p ≤ 0.0001. Two-way ANOVA with Bonferroni post-test. All data show mean ± SEM. [file mmc2.pdf]
